# Update on the Pathogenesis, Clinical Diagnosis, and Treatment of Hirayama Disease

**DOI:** 10.3389/fneur.2021.811943

**Published:** 2022-02-01

**Authors:** Hongwei Wang, Ye Tian, Jianwei Wu, Sushan Luo, Chaojun Zheng, Chi Sun, Cong Nie, Xinlei Xia, Xiaosheng Ma, Feizhou Lyu, Jianyuan Jiang, Hongli Wang

**Affiliations:** ^1^Department of Orthopedics, Huashan Hospital, Fudan University, Shanghai, China; ^2^Spine Center Fudan University, Shanghai, China; ^3^Department of Neurology, Huashan Hospital, Fudan University, Shanghai, China; ^4^Department of Orthopedics, Shanghai Fifth People's Hospital, Fudan University, Shanghai, China

**Keywords:** Hirayama disease, pathogenesis, clinical manifestations, medical imaging, electromyography, diagnosis criteria, treatment

## Abstract

Hirayama disease (HD) is characterized by the juvenile onset of unilateral or asymmetric weakness and amyotrophy of the hand and ulnar forearm and is most common in males in Asia. A perception of compliance with previous standards of diagnosis and treatment appears to be challenged, so the review is to update on HD. First, based on existing theory, the factors related to HD includes, (1) cervical cord compression during cervical flexion, (2) immunological factors, and (3) other musculoskeletal dynamic factors. Then, we review the clinical manifestations: typically, (1) distal weakness and wasting in one or both upper extremities, (2) insidious onset and initial progression for 3–5 years, (3) coarse tremors in the fingers, (4) cold paralysis, and (5) absence of objective sensory loss; and atypically, (1) positive pyramidal signs, (2) atrophy of the muscles of the proximal upper extremity, (3) long progression, and (4) sensory deficits. Next, updated manifestations of imaging are reviewed, (1) asymmetric spinal cord flattening, and localized lower cervical spinal cord atrophy, (2) loss of attachment between the posterior dural sac and the subjacent lamina, (3) forward displacement of the posterior wall of the cervical dural sac, (4) intramedullary high signal intensity in the anterior horn cells on T2-weighted imaging, and (5) straight alignment or kyphosis of cervical spine. Thus, the main manifestations of eletrophysiological examinations in HD include segmental neurogenic damages of anterior horn cells or anterior roots of the spinal nerve located in the lower cervical spinal cord, without disorder of the sensory nerves. In addition, definite HD needs three-dimensional diagnostic framework above, while probable HD needs to exclude other diseases *via* “clinical manifestations” and “electrophysiological examinations”. Finally, the main purpose of treatment is to avoid neck flexion. Cervical collar is the first-line treatment for HD, while several surgical methods are available and have achieved satisfactory results. This review aimed to improve the awareness of HD in clinicians to enable early diagnosis and treatment, which will enable patients to achieve a better prognosis.

## Introduction

Hirayama disease (HD), which is also referred to as juvenile muscular atrophy of the distal upper extremities or monomelic amyotrophy, is a special neurological disorder that was first reported by a Japanese neurologist, Keizo Hirayama (1). HD is a regional and male-prone disorder that is characterized by the juvenile onset of unilateral or asymmetric weakness and amyotrophy of the hand and forearm muscles supplied by myotomes C7-T1, with sparing of the brachioradialis muscles and no objective sensory disturbance or lower limb involvement.

Most HD patients have characteristic abnormal forward-shifting of the posterior dura (crescent-shaped loss of attachment behind the posterior dura) during neck flexion on magnetic resonance imaging (MRI), and both autopsy and neuropathologic studies have demonstrated that major lesions of HD occur primarily in locations such as the cervical anterior horn and ventral roots. Therefore, restricting neck flexion (e.g., neck collar support and surgical treatment) has become the main method of treating HD. More importantly, an increasing number of studies have demonstrated that this benign disease, if not treated early and reasonably, may cause serious dysfunction with loss of productivity (2–7). However, both neck collar support and surgical treatment have been applied in only a small number of cases due to the lack of awareness of this disease, although clinician-led guidelines on the diagnosis and treatment of HD were published in 2020 (8).

The names given to the entity herein described as Hirayama disease vary, and it is urgent to standardize the name to make it possible for physicians to identify the disease category. To our knowledge, there exist 14 different names describing the entity. We classified these into six distinct types to better understand them. First, some names containing “distal,” for instance, *juvenile muscular atrophy of (a) distal upper extremity, benign juvenile muscular atrophy of the distal upper extremity, distal bimelic amyotrophy, juvenile amyotrophy of the distal upper extremity*, and *segmental muscular atrophy of distal upper extremity with juvenile onset*, are incomplete descriptions due to the presence of proximal alone or combined distal atrophy in a great number of cases; the use of such names will mislead clinicians, likely leading to misdiagnosis or delays in diagnosis (1, 9–12). Second, *monomelic amyotrophy* (MMA) is a broader concept that includes crural MMA and brachial MMA; the latter includes other diseases that are characterized by atrophy of the upper limbs (13). It could be more appropriate to use the name “MMA” for similar cases that do not meet the characteristics and pathogenesis of HD and do not display other precisely defined disorders (10). Third, another term, *benign focal amyotrophy*, similar to MMA, involves either proximal or distal portions of the upper or lower extremities, with no compressive or traumatic etiology and arising later in life. Therefore, benign focal amyotrophy encompasses a more heterogeneous group of amyotrophies (14). Fourth, some names that include the term “spinal muscular atrophy” (SMA), for example, benign *juvenile brachial SMA, distal SMA*, and *juvenile asymmetric segmental SMA*, are not appropriate, for the reason discussed above (9–11). Fifth, *cervical flexion (induced) myelopathy* might be a suitable name for the entity, but it has been used relatively rarely in studies (15). We recommend that the condition continue to be designated “Hirayama disease,” or juvenile benign muscular atrophy of upper extremity, according to current knowledge of the entity. Because, as adapted from the statements of Haluk Yavuz et al., HD is a type of juvenile muscular atrophy of the upper extremity with proximal, distal, and proximodistal involvement due to radiologically proven and arrested flexion myelopathy without sensory disturbance or with minimal sensory disturbance (10). From the perspective of pathogenesis that cervical flexion plays an important part in, it may be also called as juvenile cervical flexion myelopathy properly.

In the present study, we mainly review the studies published in the last 10 years, including previous clinician-led guidelines, and report the current strategies for diagnosis and management of HD.

## Epidemiology

HD was first reported in 12 cases by Hirayama in 1959, who termed it “juvenile muscular atrophy of the unilateral upper extremity.” In 1982, the hypothesis was proposed that HD is caused by the compression of the lower cervical spinal cord as a result of an imbalance of the distance between the spinal cord and the vertebral canal (16). Importantly, in 1987, the first autopsy of a patient with HD was performed, which revealed anterior horn cell (AHC) shrinkage that was most severe at C7 and C8, indicating that HD is a disease of AHCs rather than a subtype of motor neuron disease (MND) (17).

In 2006, Tashiro et al. conducted a nationwide survey of patients with HD in Japan that included information on epidemiology, period of disease progression, neurological, neuroradiological, electrophysiological and cerebrospinal fluid findings, and treatment. This is the only complete epidemiological survey that has been completed to date (18). Most HD patients are from Asia. During the last two decades, more than 900 patients with Hirayama disease have been included in clinical studies conducted in mainland China (19–23). There have been reports of increasing numbers of cases in other countries and regions including India, North America, Europe, and Australia (3, 24–27). Inadequate understanding of the disease by pediatric neurologists in North America may decrease the rate of diagnosis of HD, and there is no doubt that the number of HD patients has increased (26). In studies of HD, the reported male-to-female ratio varies: 8.3:1 (281 males to 34 females) and 20:1 in Japan; 2.8:1 in India; and 11:1 (67 males to 6 females) and 31.6:1 (348 males to 11 females) in China (18, 20, 28–30). The concealment of onset in females may also be the cause of the inconsistency of onset between males and females. Most patients suffer from HD in their late second or early third decades, predominantly beginning at the age of 17–19 (18).

## Pathogenesis

In early studies, HD was recognized as a subtype of SMA and described as “juvenile asymmetric segmental spinal muscular atrophy” because the clinical manifestations were similar to those of SMA. However, genetic studies demonstrated that the form of survival motor neuron gene deletion found in patients with SMA does not occur in HD patients (31), and significant cervical structural abnormalities and the relatively good clinical prognosis of HD patients further highlighted the difference between the two diseases. Similarly, unlike patients with amyotrophic lateral sclerosis (ALS), patients with HD also present with no superoxide dismutase 1 gene deletion (32). Furthermore, through whole-exome sequencing, Lim et al. found that only two variant genes, KIAA1377 and C5orf42, are associated withHD. Thus, clinicians established HD as a new entity that differs from SMA, ALS and other MNDs (33).

Taking into account the characteristic abnormal forward shifting of the posterior dura during neck flexion in MRI, cervical cord compression during cervical flexion was considered one of the possible pathogenic conditions leading to HD. (1) Dural sac dysplasia: dural sac dysplasia could arise from the disproportionate lengths of the spinal cord and the spinal canal due to the growth spurt that occurs during puberty, leading to tightness of the spinal dura mater; this is why HD is self-limiting after puberty, and the difference in the growth rates of males and females also explains the predominance of HD in males (34–36). The anterior shifting of the tight spinal dura mater during neck flexion causes the cervical spinal cord to oppress the posterior portions of the cervical vertebrae (37). The resulting compression leads to deficits in circulation through the spinal anterior artery, and infarctions involving the AHCs in C7-T1 occur (38–40). (2) Nerve root dysplasia: the nerve root dysplasia observed in patients might be related to the short length of the cervical roots. During neck extension, slackness of the dorsal roots disappears on the affected side, on which the arm grows faster than on the other side. On neck flexion, the right dorsal roots cannot extend, and this causes the cord to be drawn anteriorly to the affected side (41). (3) Structural abnormalities of the spinal ligament: the tightness of the posterior dura mater may also be caused by loss of fibrous connective tissue, including loss of both elastic fibers and the normal wavy structure of the dura and loss of the epidural ligament (42, 43). Asymmetry of the epidural ligaments is the reason why atrophy is usually unilateral or asymmetric. (4) Venous dysplasia: engorgement of the venous plexus is considered another possible cause of compression of the spinal cord. There are three mechanisms through which the venous plexus engorges: negative pressure in the posterior epidural space due to anterior displacement of the dura; impaired venous drainage in the jugular veins during cervical flexion and consequent diversion of flow toward the posterior epidural plexus; and shifting of blood to the posterior epidural compartment from the compressed anterior venous plexus (44). Importantly, both autopsies and neuropathologic studies have demonstrated that major lesions of HD occur primarily in locations such as the cervical anterior horn and the ventral roots. Therefore, ischemic injury of the cervical anterior horn and/or nerve root caused by excessive forward displacement of the posterior dura during neck flexion has become the main current hypothesis regarding the pathogenic mechanism leading to HD.

Some studies have shown that immunological factors may contribute to the pathological process. Kira et al. reported a high frequency of coexistent atopic disorders, hyperIgEaemia, and mite antigen-specific IgE, together with a family history of allergic disorders, in their patients, strongly suggesting the presence of an atopic tendency in HD (45). Ito et al. found that hyperIgEaemia was related to severe disabilities in HD patients and that there might be a negative correlation between IgE levels and the duration of disease progression (46). In addition to IgE, Petrova et al. found that IgA deficiency may contribute to vulnerability of the spinal cord to ischemia in HD patients (47). Anti-GQ1b antibody, an antiganglioside antibody that has also been found in patients, appeared not to be reliable for HD diagnosis and unsuitable as either a diagnostic or a prognostic factor (48). To our knowledge, all immunological studies related to HD have been retrospective studies of small sample groups; therefore, we are unable to assess the causality between immunological factors and outbreaks of HD.

In recently published studies, Khadilkar et al. reported that their patients had longer necks than did normal patients (49). Li et al. found that the imbalance between the strengths of flexor and extensor muscles that was found in HD patients during cervical flexion resulted in dynamic instability and showed that it was a risk factor for spinal cord atrophy. However, all of the above findings need to be confirmed in future studies (50). Wang et al. found that the neutral and flexion Cobb angles in HD patients decreased from C2/3 to C5/6 but increased at C6/7; this may be another cause of the observed dynamic instability (51).

## Clinical Manifestations

### Typical Clinical Manifestations

**Figure 1:**
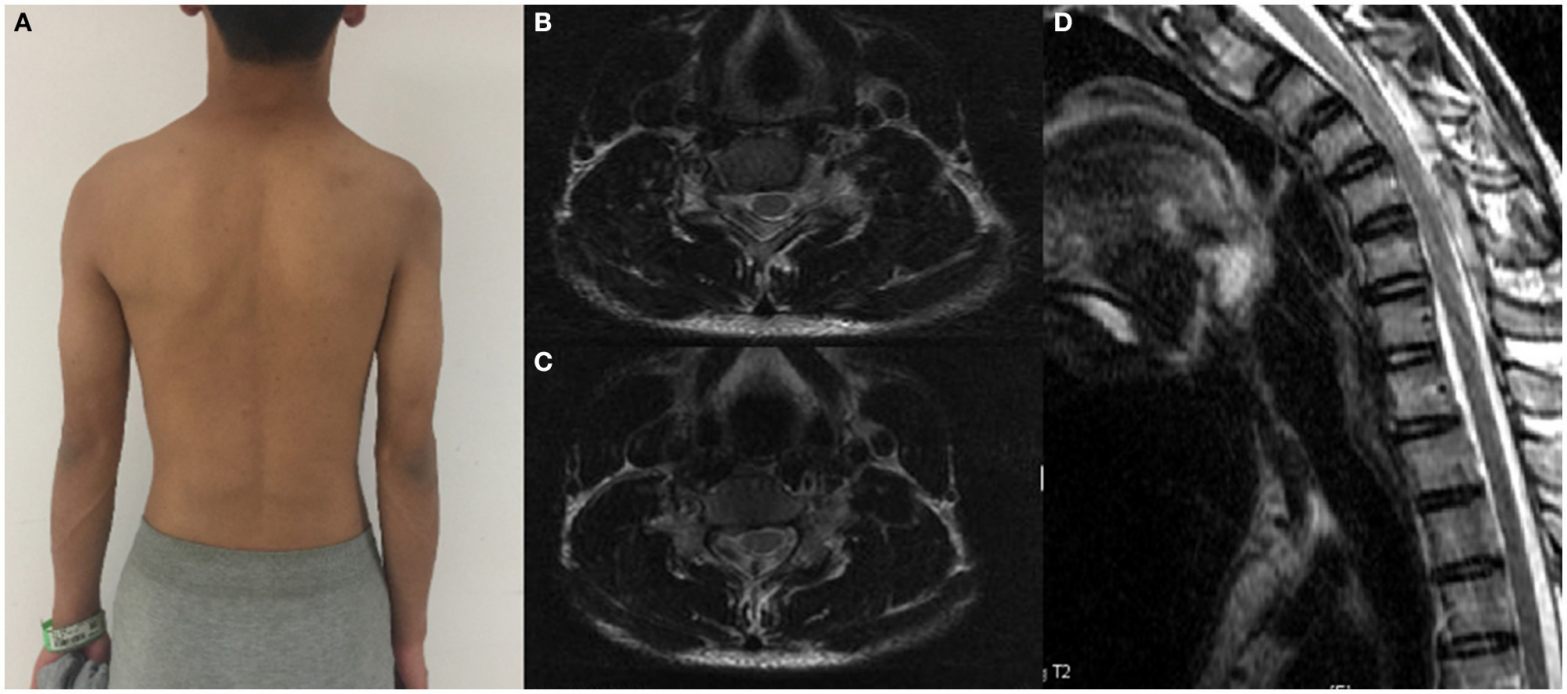
A patient with significant atrophy of the right proximal muscles of the upper extremity and his cervical flexion MRI. **(A)** Proximal muscle atrophy of right upper extremity. **(B,C)** Spinal cord flattening in horizontal view. **(D)** LOA in sagittal view.

Since HD was first reported, clinicians have continuously updated their understanding of the clinical manifestations of the disease. In the past 10 years in particular, the continuous improvement in clinicians' awareness of the disease and the numerous relevant studies have enabled the compiling of the common clinical manifestations of HD (1, 8, 18, 23, 29), which comprise five primary symptoms and manifestations: (1) distal weakness and wasting, predominantly on the ulnar side, in one upper extremity or asymmetrically in both upper extremities. Most patients show more severe muscle atrophy on the ulnar side, a condition termed “reverse split hand syndrome,” which may be a feature that distinguishes HD from ALS. The AHCs that innervate the hypothenar muscles are most susceptible to this type of damage, whereas death due to apoptosis appears to more prominently affect the AHCs that innervate the radial muscles in ALS (52); (2) insidious onset and initial progression for 3–5 years, followed by arrest of the disease or a relatively benign period (1). (3) irregular, coarse tremors in the fingers of the affected hand(s); (4) mild transient worsening of symptoms when the individual is exposed to a cold environment. A previous study reported that cold paresis was observed in 97% of HD patients (53), patients with cold paresis have a longer strength-duration time constant and higher refractoriness than patients without cold paresis. It is possible that cooling the nerve causes a decrease in sodium-potassium adenosine triphosphant activity, which results in transient depolarization due to the inactivation of the electrogenic sodium pump (54); (5) absence of objective sensory loss (Figure 1).

The key problem with the above list is that an increasing number of cases with atypical symptoms are diagnosed by MRI or EMG. In such cases, we should recognize and intervene to avoid poor prognosis due to misdiagnosis and delay in treatment. With the progression of the disease, these symptoms will appear when HD reaches a severe stage rather than remaining absolutely atypic.

### Atypical Clinical Manifestations

#### Pyramidal Signs

HD is considered a disease of AHCs, but pyramidal signs, especially the Hoffmann sign, appear in many patients. It was found in 2.4% of patients in Japan and 10.6% in China (18, 29). The pyramidal sign is assumed to be a consequence of a severe degree of spinal cord injury, and whether or not the pyramidal tract shows disorders depends on the severity of HD (6, 55).

#### Atrophy of the Muscles of the Proximal Upper Extremity

**Figure 2:**
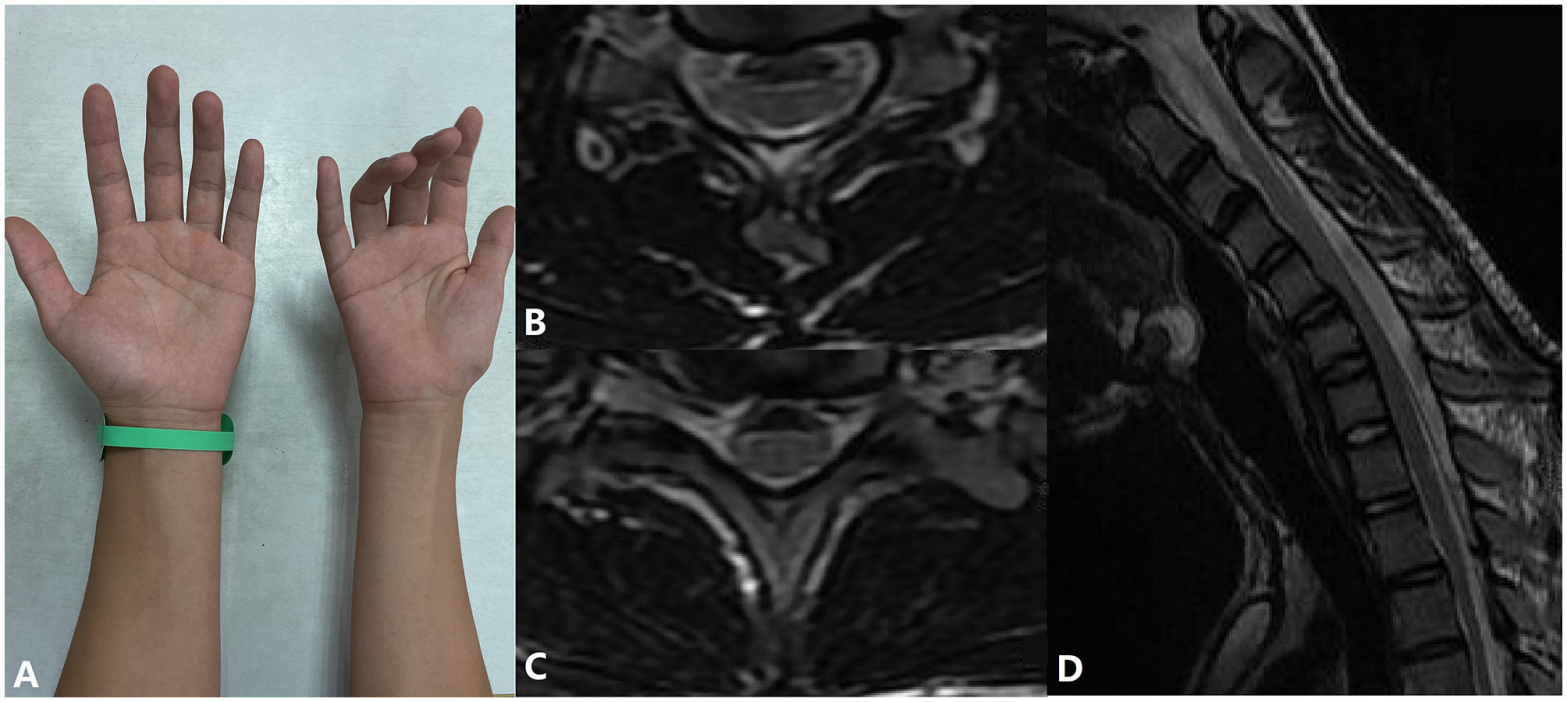
A patient with atrophy of the muscles of the right hand and forearm and his cervical flexion MRI. **(A)** Inability to extend fingers and muscle atrophy in the patient's right hand. **(B,C)** Spinal cord flattening and LOA in horizontal view. **(D)** LOA in sagittal view.

The compression associated with HD is usually restricted to the lower cervical spinal cord, mainly at the C7-T1 level; thus, the atrophy observed in most patients is located in the distal upper extremity. However, in not a few reported cases the symptoms emerge proximally, and some of these patients were subclinical cases (13, 18, 32) (Figure 2). Preethish-Kumar et al. showed that distal, proximal, and proximodistal bimelic amyotrophy are all myelopathies that can be induced by cervical flexion (40). Thus, the functional outcome of proximal forms is likely worse and more uncertain than that of distal forms (14, 56).

#### Long Progression

In the majority of HD patients, the progression of the disease is self-limiting, where progression occurs over <5 years. However, there have indeed been cases in which the disease progressed over more than 10 years, where the patient's condition continued to deteriorate after a stable period (44). The changes observed on imaging do not improve as the disease progresses, which indicates that HD may not be a self-limiting disease in some cases (57).

#### Sensory Deficits

A number of patients with sensory deficits have been reported. These deficits occur because compression injures the tracts that conduct information from the sensory organs, and the deficits likely become more severe due to higher and higher stress (25). According to Tashiro et al., 64 of 333 (19.2%) HD patients had sensory deficits (18).

Importantly, the clinician-led guidelines have added some of these symptoms to the list of symptoms that should be considered in the diagnosis of HD, and as physicians, we should reevaluate our stereotypes (8). First, some patients with HD may experience disease progression after an interval in which the disease remains at a stable stage. Second, in some patients with HD, the duration of the disease may be more than 5 years. Third, HD allows the presence of clinical symptoms (including atrophy, weakness, and denervation) of symmetrical functional involvement of both upper limbs. In addition, a small number of patients with HD may experience subjective paresthesia in the upper limbs during the early stage of the disease. Finally, pyramidal tract signs can be present, in a certain percentage of patients.

## Imaging Manifestations

Almost all HD patients show straight alignment or kyphosis of the cervical spine; this is common but not specific to HD. Myelography shows forward movement of the posterior dural wall during neck flexion. According to Chen et al., some signs that appear on neutral-position MRI require our attention (58). These are (1) asymmetric cord flattening and localized lower cervical cord atrophy due to compression by the tight posterior dural wall, mostly at the C4 to C7 level; (2) abnormal cervical curvature (straight or kyphotic cervical spine alignment); (3) loss of attachment between the posterior dural sac and the subjacent lamina (however, this is considered a neck-flexion MRI sign in some studies because it also manifests in normal people when they are in the cervical neutral position) (59); (4) non-compressed intramedullary high signal intensity on T2-weighted imaging (T2WI), a sign that can be caused by ischemia or necrosis of AHC. A symmetrical bilateral high-signal-intensity lesion on axial T2WI is found in some HD patients; this sign has been termed “snake-eye appearance” because it resembles the face of a snake. Furthermore, the presence of snake-eye appearance indicates an irreversible lesion, a poor prognosis and the need for timely surgical intervention (19).

Neck-flexion MRI is considered the most important imaging examination for the diagnosis of HD; it reveals the pathogenesis of HD and compression of the lower cervical spinal cord due to the abnormal tightness of the dural sac during cervical flexion. Signs include forward displacement of the posterior wall of the cervical dural sac, cord flattening, atrophy, and the presence of a crescent-shaped high-intensity mass.

In HD, lower cervical cord flattening and atrophy are present in the neck-flexion MRI, similar to the neutral position (30, 60). Another hallmark sign is the forward displacement of the spinal cord due to the presence of a shorter dural sac. The disproportionate distance between the vertebrae and their contents due to the juvenile growth spurt and the suspended dura mater anchored only at C2 to C3 and coccyx result in tightness of the dural sac during neck flexion, causing forward displacement of the posterior wall of the cervical dural sac followed by forward displacement of the cervical spinal cord and cord flattening. Consequently, microcirculatory disturbances in the region served by the anterior spinal artery caused by long-term compression bring about ischemia and necrosis of AHCs; as a result, the cord appears thin due to atrophy. In addition, a crescent-shaped high-intensity mass with curvilinear flow-void signals inside it appears in the posterior epidural space during neck flexion. The mass disappears when the neck returns to its normal position, revealing congestion of the venous plexus rather than vascular malformation or tumors (61). The congestion of the venous plexus is due to the three factors discussed above. Postgadolinium T1 fat-suppressed images show an enhanced posterior epidural venous plexus with flow voids within it at the affected levels of the spinal cord and asymmetric flattening of the affected hemicord (62). Furthermore, forward displacement of the posterior wall of the cervical dural sac, LOA, and the presence of a crescent-shaped high-intensity mass are different imaging manifestations of the same cause.

All signs detected on neck-flexion MRI indicate the pathogenesis of HD and correspond to clinical manifestations. Regardless of the severity of the patient's symptoms and the neutrality of the position MRI shown in the MRI, neck-flexion MRI is required to obtain a definite diagnosis of HD. Although there are no optimal flexion requirements for cervical-flexion MRI, a cervical flexion angle of 35° is recommended to achieve the best appearance and an accurate diagnosis (37).

Diffusion tensor imaging (DTI), a functional imaging technique, is based on the Brownian motion exhibited by water molecules in tissues and can potentially be utilized in the diagnosis of HD (63) A higher apparent diffusion coefficient (a measure of the magnitude of water molecular diffusion) and lower fractional anisotropy (the fraction of the total diffusion that is measured in one specific voxel) are potential diagnostic parameters for HD in DTI (64). DTI is used not only to evaluate spinal cord injury and the prognosis of spinal cord diseases involving HD but also to choose the level at which to operate. Functional imaging is an important issue for future research, and further work is required to establish the usefulness of DTI in diagnosing HD accurately and as early as possible. Blood oxygenation level dependent-functional MRI of HD patients showed significant ipsilateral activation more frequently in symptomatic hand tasks than in asymptomatic hand tasks, and activation of both the contralateral and ipsilateral primary motor areas was stronger during symptomatic hand tasks. In addition, the activation was reduced after surgery (65).

## Electrophysiological Examinations

Electrophysiological examinations are important for the diagnosis and differentiation of HD. The main manifestations of EMG include segmental neurogenic damage of the AHCs or anterior root of the spinal nerves located in lower cervical spinal cord, without disorders of the sensory nerves. EMG is commonly used in the neurological examination of HD patients. It shows neurogenic lesions in patients in whom the disease has been present for a short period of time (these are indicated by fibrillations and positive sharp waves) and decreased motor unit number estimation (MUNE) and large potentials (due to denervation and re-innervation) in patients in whom the disease has been present for a longer period (66). The presence of spontaneous potentials indicates that the disease is in the progressive phase (20, 67). More importantly, cases with subclinical unilateral symptoms on the affected contralateral side were found, perhaps reminding us that the illness will eventually affect the upper extremities bilaterally if the intervention is not effective or does not occur in time (20).

Motor nerve conduction studies have shown decreased amplitudes and delayed latencies in the affected upper limbs muscles without abnormal conduction velocities (68). The ulnar/median compound muscle action potential ratio is decreased in HD patients, which is consistent with symptoms that indicate that the ulnar side of the affected limb is more severely affected. In MND patients, an increase in the ulnar/median-ratio has been reported. Sensory nerve conduction studies typically yield normal results in HD patients, which correspond to the clinical manifestation and pathogenesis of the disease (20, 69). Thus, generally, conduction velocities do not change, although if abnormal conduction velocities are found, peripheral nerve lesions should first be considered.

F waves in HD patients show decreased frequency and conduction velocity but normal latency (20). F-wave studies in HD patients have yielded varied result: Hassan et al. found that F-wave conduction velocities normal in HD, whereas Zheng et al. reported a delay in F-wave latency in HD (68, 70). The latter study reported a significant increase in the percentage of median repeater F waves, which may be attributed to reinnervation by a distal (and/or proximal) axonal sprouting mechanism or supramaximal stimulation, which preferentially activates large motor neurons that produce the F wave during neck flexion (70). Moreover, repeater F waves were observed, the percentage of ulnar and median repeater F waves significantly increased, and higher average peak-to-peak amplitude of F-waves expressed as a percentage of the maximal M-wave amplitude were observed on the symptomatic side during neck flexion, compared with during a neutral posture., which supports the idea that remnant AHCs are inhibited during cervical flexion. Delayed F-wave latency, reduced conduction velocity, and lower persistence are expected in some cases of cervical spondylotic myelopathy, whereas normal or lower persistence is expected in MND, which helps practitioners distinguish between these conditions. Results of studies that examined somatosensory evoked potentials (SEP) and motor evoked potentials (MEP) in HD patients vary considerably. Abraham et al. found that the latency of SEPs was normal in HD, whereas Park et al. reported that the N13–N20 interpeak latency during neck flexion was significantly correlated with the presence of HD (71, 72). For MEP, decreased amplitude, longer central motor conduction time, and delayed latency have been observed in HD patients, as well as a significant reduction in the upper limb amplitude of MEPs on cervical stimulation compared with that in the neutral position, although conclusions drawn in several studies are inconsistent (71, 73). In contrast, SEPs are not show any significantly different between the neutral and cervical positions in HD patients (71, 73). Recently, it was found that the amplitude of intraoperative MEP increases during surgery, and this persists until the end of the surgical procedure. Therefore, the amplitude of intraoperative MEP may help define an adequate fixation position (74).

MUNE is a quantitative method that can be used to estimate the number of motor units innervating a muscle or a muscle group. This provides a direct index through which to measure the severity and progress of HD and the effects of treatment. MUNE values are negatively correlated with illness duration, and a decreased MUNE value in a patient with normal muscle strength indicates that the MUNE value changed before the symptoms emerged (75). The motor unit number index also shows subclinical changes that appear as a decrease before muscle strength changes (76).

## Diagnosis and Classification

### Diagnostic Criteria

Recently, numerous reports have shown that HD patients exhibit the pyramidal sign, a long period of disease progression, and sensory deficits, which suggests that the diagnostic criteria above are no longer suitable for the current clinical understanding of HD. Previously, in clinical practice, we found that when clinical manifestations are used as the primary basis for diagnosis, diagnosis is delayed. However, it is now widely recognized that medical imaging and EMG, especially neck-flexion MRI, are of significant value in the diagnosis of HD. Thus, it was necessary to update HD diagnostic criteria, which led to the development of the Huashan diagnostic criteria for HD. These criteria take into account clinical manifestations, medical imaging manifestations, and electrophysiological examinations, based on recent reports and our experiences (29). The details are as follows (Table 1).

**Table 1:** The Huashan diagnostic criteria for Hirayama disease (29).

	**Clinical manifestations**	**Imaging manifestations**	**Eletrophysiological examinations**
Elements for definite diagnosis	① Occult onset during puberty, more common in males	① Atrophy or thinning of the middle and lower cervical spinal cord on either neutral or flexion MRI	① Neurogenic lesions located in anterior horns and/or roots of the middle and lower cervical spinal cord
	② Localized muscular atrophy and weakness of the upper extremities, predominantly in the ulnar forearms and the intrinsic muscles of the hands unilaterally or mainly on one side	② LOA or the presence of a crescent-shaped high-intensity mass at the posterior epidural space on T2WI	② Normal or only mild abnormal conduction velocity in peripheral nerves of the upper limbs
	③ Absence of cranial nerve involvement and muscular atrophy in other parts of the body such as the lower limbs		③ Absence of obvious involvement of the cranial nerves and the thoracic, lumbar or sacral spinal cord
Other elements	④ Cold paralysis and tremors in fingers when they are stretched	③ Anterior displacement and flattening of the lower cervical spinal cord and narrowing or absence of the anterior spinal space on neck flexion MRI	
	⑤ Active deep tendon reflex and/or positive pathological signs in parts of patients	④ High-intensity signs located in the anterior horn areas on T2WI in parts of patients	
	⑥ Mild sensory deficits in the upper limbs in a small number of patients	⑤ Straight alignment or kyphosis of the cervical spine in X-rays in parts of patients	
Definite HD^*^	Meeting criterion ①, ②, and ③	Meeting criterion ① or ②	Meeting criterion ①, ② and ③
Probable HD^**^	Meeting criterion ② and ③		Meeting criterion ③

* *Definite HD means meeting criterion above, with or without other elements*.

** *Probable HD means meeting criterion above and lack of 1–5 other elements for definite diagnosis, with or without other elements*.

Definite HD refers to individuals with all ①–③ *elements for definite diagnosis* in both “clinical manifestations” and “electrophysiological examinations”, and one of the two *elements for definite diagnosis* in “imaging manifestations”, with or without some of the *other elements*. Probable HD is defined by: meeting criterion ②, ③ in “clinical manifestations” and criterion ③ in “electrophysiological examinations”, but missing 1–5 of the other *elements for definite diagnosis*, with or without some of the *other elements*.

In brief, definite HD needs 3-dimensional diagnostic framework, while probable HD needs to exclude other diseases *via* “clinical manifestations” and “electrophysiological examinations”.

Compared the new diagnostic criteria with diagnostic criteria proposed by Hirayama (1), “progressive aggravation within 3–5 years after onset” is not mentioned, and localized muscular atrophy of the distal upper limbs, lack of sensory disturbance, and lack of pyramidal tract signs are no longer emphasized. The new criteria specify a clear move forward of the cervical spinal cord, flatten of the spinal cord under MRI during neck flexion, a crescent-shaped hyperintensity shadow (LOA) on T2WI, and segmental nerve injuries limited to the lower cervical segment and appearing mostly in segments C7/8–T1.

### Differential Diagnosis

HD is a local abnormality of the cervical spine, with atrophy and weakness of the upper limb muscles as the main clinical manifestations, which lead to neurological dysfunction. Therefore, it is necessary to perform a differential diagnosis from common diseases that cause muscular atrophy of the upper limbs, such as ALS-related diseases, cervical spondylosis, peripheral nerve compression syndrome, (e.g., carpal tunnel, cubital tunnel, supinator, and thoracic outlet syndromes), syringomyelia, intramedullary neoplasm, and ossification of the posterior longitudinal ligament of the cervical spine.

Using age of onset, alongside cervical MRI, computed tomography, and other imaging examinations, HD can be clinically distinguished from syringomyelia, intramedullary neoplasm, and ossification of the posterior longitudinal ligament of the cervical spine. Moreover, upper limb peripheral nerve compression diseases can be excluded with electrophysiological examinations.

Cervical spondylotic amyotrophy and ALS can be easily confused with HD in the early stage of the disease (8, 77–79). Clinical identification needs to be considered from numerous perspectives, such as age of onset, clinical manifestations, and imaging and electrophysiological examination results (Table 2).

**Table 2:** Clinical differential diagnoses among HD, cervical spondylotic amyotrophy, and ALS (8, 77, 78).

**Points**	**HD**	**Cervical spondylosis amyotrophy**	**ALS**
Age of onset	Puberty, 12–20 years old predominantly	Middle-aged and elderly people, 40–60 years old predominantly	Middle-aged and elderly people, 40–60 years old predominantly
Course of illness	Insidious onset, with a plateau after 3–5-year progression	Long course, with slow progression	Insidious onset, continuous progression, and death after 3–5 years
Pathology	Damages of the anterior horns or (and) the anterior nerve roots of the cervical spinal cord caused by cervical flexion	Compressions of the anterior horns or nerve roots of cervical spinal cord caused by cervical degeneration	A group of chronic progressive neurodegeneration that mainly damages the anterior horns of the spinal cord, cranial nerve motor nuclei and pyramidal tracts
Atrophic muscle	Mainly the hand inner muscles, the forearm muscles affected usually	Mainly deltoid and biceps in the proximal type; dominantly hand inner muscles in the distal type	Only the localized muscles of any limb maybe affected in the early stage; and muscles of the limbs, even the neck, tongue, and throat muscles maybe affected gradually in the late stage
Sensory deficits	There is no hypoesthesia generally, and a small number of patients with a long course may complained mild sensory deficits in the upper limbs	Most cases have no or only slight sensory deficits, and those with a long course of illness may have sensory deficits in limbs with different degrees	There is no sensory deficits generally
Muscle weakness	The distal muscles of the upper limbs mainly	Muscles of proximal or distal upper extremity depending on different types	Localized muscles of a single limb in the early stage; and a wide range, involving muscles of the limbs, oropharynx, and respiratory muscles in the late stage
Deep tendon reflex	Generally normal or mildly decreased reflexes in the upper limbs; and active or hyperactive reflexes of lower limbs parts of patients	Generally normal or mildly decreased reflexes in affected upper limb; and active or hyperactive reflexes of lower limb in patients with a long course	Active or hyperactive reflexes in all extremities
Pyramidal signs	Generally negative, but positive in parts of patients	Positive in patients with long course of illness	Generally positive
Electrophysiological examination	Damages to the anterior horns and/or anterior roots of the middle and lower cervical spinal cord, and asymmetrically generally	Damages to the anterior horns and/or anterior roots of the middle and lower cervical spinal cord, and asymmetrically generally	Atypical in the early stage of the illness, and damages to the nerves in multiple regions (cranial, cervical, thoracic, lumbar, and sacral) in the late stage

### Clinical Classification

With the advances in clinical understanding of HD, it has been found that there is considerable variation in the clinical manifestation and severity between HD patients. Moreover, whether HD patients are in the advanced or stationary stage significantly influences the choice of intervention options; thus, appropriate clinical classifications must be adopted. As such, several scholars have attempted to classify HD into three types, to classify and evaluate HD patients (29, 80). Type I patients display atrophy of the inner muscles of the hand and forearm in the upper limb unilaterally or mainly on one side. Depending on whether the symptoms have progressed and whether electrophysiological examinations have been conducted in the past 6 months, type I can be further divided into a stable period (subtype Ia) or a progression period subtype (subtype Ib). Type II patients exhibit atrophy of the inner muscles of the hand and forearm in the upper limb unilaterally or mainly on one side, as well as pyramidal tract injury (active or hyperactive knee reflex, and positive Hoffmann signs). Type III represents atypical HD, in which there may be atrophy of the proximal muscles of the upper limb, symmetrical and bilateral muscle atrophy of the upper limbs, and sensory disturbances associated with upper limb numbness (Table 3). Patients whose symptoms fit more than one classification should be preferentially assigned the higher-level classification.

**Table 3:** The Huashan clinical classification system for HD and the suggestions of treatment (29, 80).

**Type**	**Clinical manifestations**	**Progression in the past 6 months**	**Suggestions for treatments**
I	Hand inner muscle and forearm muscle atrophy in unilateral upper limb or asymmetrical bilateral upper limbs	Ia: stable period	Regular follow-up assessment was recommended. If the disease progressed, to wear a cervical collar was suggested; surgery could be done if necessary.
		Ib: progression period	It was recommended to wear a cervical collar, and evaluate regularly. If patients could not wear cervical collar for long, it was recommended to operate.
II	Hand inner muscle and forearm muscle atrophy in unilateral upper limb or asymmetrical bilateral upper limbs with pyramidal tract injury	–	Surgical treatment was recommended
III	Atypical HD, including upper limb proximal muscle atrophy, amyotrophy of symmetrical bilateral upper limbs, and sensory deficits with upper limbs	–	Wear a cervical collar, and follow-up and assess closely, and choose surgical treatment if necessary

## Treatment

### Non-surgical Treatment

Although HD is self-limiting, it is necessary to reach an early diagnosis and intervene effectively. Non-surgical treatment using cervical collars is the first-line treatment for halting the progression of the illness in many patients; this method prevents the further progression of HD by restricting neck movement and preventing compression injury to the spinal cord during flexion (81). Cortese et al. found that the venous plexus decreased in size after this treatment (82). According to Rajeev et al. and Rajesh et al., subjective muscle strength increases after the use of cervical braces (83, 84). Narayana et al. reported that cervical collar treatment was effective for patients with Hirayama disease (85). Tokumaru et al. evaluated the therapeutic effects of the use of cervical collars by 38 patients with Hirayama disease by comparing them with 45 patients with untreated Hirayama disease (81). The results showed that the duration of the disease decrease with cervical collar treatment, and 15 patients who were within 2.5 years of onset even experienced relief of muscle weakness and cold paralysis, suggesting that cervical collar treatment is effective. At the same time, early diagnosis is very important, and it was pointed out that early diagnosis and clinical intervention might reduce the dysfunction that typically develops in adolescent patients (82). A shorter disease duration compared to the untreated group was also reported in numerous studies. Nonsurgical treatment is still the most commonly used treatment in HD patients.

There are several problems with the use of cervical collars. First, the collar must be worn for 24 h daily for 3–4 years, and this could be unbearable to a high number of patients (86, 87). Second, in some cases, the cervical collar will halt the progression of the disease but will not improve the objective indicators due to the decrease in MUNE (88). The reason why muscle strength increases after treatment is that the reinnervation process can maintain strength (89). Third, a few reports show that the disease can worsen during a period of conservative treatment (3). The results would probably be irreversible if significant deterioration occurred while patients were receiving treatment with a cervical collar.

In addition, it has been shown that drugs are ineffective (90). Exercise therapy aimed at strengthening the cervical spine muscles is widely applied to improve cervical spine function and has been proven to be beneficial (91).

### Surgical Treatment

A study of 73 HD patients showed that nearly 80% of patients wore it for less than 6 months, indicating poor compliance with wearing a neck brace (92). Therefore, in recent decades, surgical procedures have been performed. The effects of surgery include not only subjective symptom relief but also improvement on objective examination. On the one hand, surgery shortens the natural duration of the illness, as shown by the clinical signs and EMG, lack of recurrence of venous congestion, and improved grip strength attributed to the recovery of muscles other than the atrophic intrinsic muscles (3, 21). On the other hand, intraoperative EMG showed immediate changes with surgical intervention, including variations in abnormal F waves and MEP (74, 93). At the same time, some improvements visible on imaging also appeared after the surgeries, including reduced range of cervical flexion motion and angle mobility of the cervical spine and increased cervical lordosis and spinal cord area (21). The idea that surgery plays an important role in the treatment of HD is agreed upon by experts in various fields (8, 21).

Surgical treatment is effective; the indications for surgery might include (1) progressive course and intolerance of the cervical collar; (2) ineffectiveness of conservative treatment; and (3) the presence of serious symptoms, a serious degree of spinal cord atrophy, the occurrence of spinal cord atrophy in rare segments, and positive pyramidal signs (8, 29).

Various surgical approaches have been utilized in clinical practice during the last two decades. These surgical approaches include laminectomy, duraplasty, corpectomy, discectomy, decompression, and fusion (3, 6, 21, 34). One or more of these approaches are selected. The procedures used can be divided into two categories: posterior approaches and anterior approaches. Posterior approaches include multilevel posterior cervical facetal fixation, short-segment posterior cervical fixation without vertebra fusion or removal of the internal fixation, cervical laminectomy and micro-resection of the posterior venous plexus, and posterior lateral mass screw fixation without decompression or fusion but with removal of the internal fixation after four years of follow-up. Anterior approaches include anterior cervical fusion or anterior cervical discectomy and fusion using intervertebral disc fusion polyetheretherketone cages or autogenous iliac bone placement (3, 21, 88, 94, 95). Various scholars have reported on the fusion of segments 1–4 based on different considerations (5, 21, 88, 96).

Early surgical treatment may not only prevent the progressive loss of motor units but also restore motor units that have undergone functional inhibition and incomplete necrosis in some HD patients (93). However, a meta-analysis showed that it is still unclear what type of surgery produces optimal results (97). Not all patients benefit from surgery, and caution should be employed when selecting patients for surgery (98).

## Prognosis

Most HD patients are given a benign prognosis for 3–5 years, followed by arrest of the disease (8). The management of cervical collar immobilization has been shown to be effective in 57.2% of cases (18). Furthermore, 46.3–87.6% of cases who accepted surgery reached an ideal stage at follow-up (34, 97, 99–101). Nervertheless, there still exist a few cases have a poor prognosis whose spastic gait persists after treatment due to non-timely treatment (2). Moreover, some patients experience atrophy or weakness of the upper limb again following a stable period (102).

## Conclusion and Future Direction

HD is a rare benign disease characterized by muscular atrophy of the unilateral or asymmetric bilateral upper extremities. It predominantly affects juvenile Asian males. As cases have been reported all over the world and research on this disease has been conducted at a rapid pace during the last two decades, some novelties need to be discussed. These problems have received a great deal of attention.

The pathogenesis of HD is still unknown, but most scholars believe that it is related to a mismatch between the length of the spinal cord and that of the spinal canal that occurs due to a growth spurt. Neck flexion MRI is the main auxiliary examination for HD; on such examination, affected individuals show spinal cord flatness, atrophy, LOA, and T2WI hyperintensity. Electrophysiologically, HD is mainly characterized by neurogenic damage without abnormal nerve conduction velocity. In terms of treatment, long-term cervical braces are the mainstay. For individuals who cannot tolerate a cervical brace or who experience short-term disease progression, surgical treatment is feasible, but the optimal surgical treatment has not been identified. The entity needs to be recognized by clinicians.

In the future, further research on the pathogenesis of HD is needed to gain a better understanding of the disease and develop more effective treatments. The surgical approach remains controversial, and thus, research prospects are broad. Additionally, a more far-reaching exploration of the optimization of the selection of surgical segments is crucial. We hope to improve clinicians' understanding of HD to allow early detection and treatment, which will offer better prognoses.

## Author Contributions

HwW and HlW conceptualized the review. HwW, YT, and JW did the literature search and analysis, and wrote original draft. CZ, SL, CS, CN, and HlW did review and editing. XX, XM, FL, JJ, and HlW were responsible for supervision. All authors contributed to the article and approved the submitted version.

## Funding

Clinical Research Plan of SHDC (No. SHDC2020CR4030), Clinical Technology Innovation Project of SHDC (No. SHDC12019X26), National Natural Science Foundation of China (No. 82072488), and AO Spine National Research Grant 2020 [No. AOSCN(R)2020-9].

## Conflict of Interest

The authors declare that the research was conducted in the absence of any commercial or financial relationships that could be construed as a potential conflict of interest.

## Publisher's Note

All claims expressed in this article are solely those of the authors and do not necessarily represent those of their affiliated organizations, or those of the publisher, the editors and the reviewers. Any product that may be evaluated in this article, or claim that may be made by its manufacturer, is not guaranteed or endorsed by the publisher.

## Abbreviations

AHCs: anterior horn cells
ALS: amyotrophic lateral sclerosis
DTI: diffusion tensor imaging
EMG: electromyography
HD: Hirayama disease
LOA: loss of attachment
MEP: motor evoked potentials
MMA: monomelic amyotrophy
MND: motor neuron disease
MRI: magnetic resonance imaging
MUNE: motor unit number estimation
SEP: somatosensory evoked potentials
SMA: spinal muscular atrophy
T2WI: T2-weighted imaging.
